# Multiscale Permutation Time Irreversibility Analysis of MEG in Patients with Schizophrenia

**DOI:** 10.3390/e27101038

**Published:** 2025-10-04

**Authors:** Dengxuan Bai, Muxuan Xue, Yining Wang, Zhen Zhang, Xiaoli Chen, Wenpo Yao, Jun Wang

**Affiliations:** 1 Institute of Intelligent Information, Hexi University, Zhangye 734000, China; baidengxuan@hxu.edu.cn; 2 College of Physics and Electromechanical Engineering, Hexi University, Zhangye 734000, China; 3 School of Chemistry and Life Sciences, Nanjing University of Posts and Telecommunications, Nanjing 210023, China; 4 Smart Health Big Data Analysis and Location Services Engineering Lab of Jiangsu Province, Nanjing University of Posts and Telecommunications, Nanjing 210023, China

**Keywords:** magnetoencephalography, schizophrenia, time irreversibility, nonequilibrium, multiscale

## Abstract

The use of questionnaire survey results as a clinical diagnostic method for schizophrenia lacks a certain degree of objectivity; thus, markers of schizophrenia in different brain signals have been widely investigated. The objective of this investigation was to explore potential markers of schizophrenia by investigating nonequilibrium features in magnetoencephalography (MEG) signals. We propose a new method to quantify the nonequilibrium features of MEG signals: the multiscale permutation time irreversibility (MsPTIRR) index. The results revealed that the MsPTIRR indices of the MEG recordings of patients with schizophrenia were significantly lower than those of the healthy controls (HCs). Moreover, the MsPTIRR indices of the MEG recordings of patients with schizophrenia and HCs differed significantly in the frontal, occipital, and temporal lobe regions. Furthermore, the MsPTIRR indices of the MEG recordings differed significantly between patients with schizophrenia and HCs in the θ, α and β bands. Abnormal nonequilibrium features mined in MEG recordings using the MsPTIRR index may be used as potential markers for schizophrenia, assisting in the clinical diagnosis of this disorder.

## 1. Introduction

Schizophrenia is a severe mental disorder characterized by various abnormalities in thinking, emotion, perception and behavior [1,2,3]. Patients with schizophrenia often experience symptoms such as hallucinations, delusions, speech and behavioral disturbances, and emotional indifference or incoherence [4,5,6]. Approximately 0.3% of the population worldwide suffers from schizophrenia, with different individuals showing varied symptoms [7,8,9]. The mechanisms underlying the pathophysiology of schizophrenia are still unknown [10,11]. The clinical diagnosis of schizophrenia is often based on the patient’s description of their behavior and questionnaire results; thus, diagnoses are subjective and lack objectivity [12,13,14,15]. Therefore, researchers have aimed to identify markers of schizophrenia in a variety of brain signals to improve the objectivity of the diagnosis of this disorder [16,17,18]. Magnetoencephalography (MEG) is highly suitable for studying schizophrenia because of its high temporal and spatial resolution [19,20].

The human brain is a very large and complex system; furthermore, the nervous system consists of several complex subsystems, and the connections between subsystems are nonlinear. Moreover, each subsystem consists of many neurons, and the interrelationships between these neurons are also nonlinear; thus, the output time series of the nervous system should have nonlinear characteristics [21,22]. Therefore, the nonlinear characteristics of the MEG signals of patients with schizophrenia can be analyzed to identify potential markers in MEG recordings, which is of practical significance for the clinical diagnosis of schizophrenia. The complexity and nonequilibrium features of complex systems are important nonlinear features that are usually measured using the information entropy and time irreversibility index of the time series [23,24]. Dynamic complex systems usually exhibit a certain degree of nonequilibrium (directionality) on the time axis, which means that the features have significantly different statistical properties when viewed in the forward and reverse directions on the time axis. This irreversible characteristic of the time series is prevalent in many practical systems, including those in physical, biological, medical, economic and social domains [24,25,26,27]. Therefore, analyzing the irreversibility indices of the output time series of complex systems is crucial to elucidate the dynamic evolution laws of these systems and provides an important basis for forecasting and decision-making.

Time irreversibility indices of electrophysiological time series have been widely used to analyze the dynamics of complex activities in physiological systems. For example, Andrzejewska et al. reported that the time irreversibility indices of electrocardiogram (ECG) signals during the QT interval were lower in patients with long Q-T syndrome than in healthy individuals [28]. Zhang et al. used an irreversibility index quantified by symbolic relative entropy and reported that energy losses in the brains of older people are significantly greater than those in the brains of younger people [29]. Moreover, Yao et al. reported that the time irreversibility index of electroencephalography (EEG) signals was significantly greater in patients with epilepsy than in healthy individuals and during seizure intervals rather than during nonseizure intervals [27]. Zanin et al. also studied the time irreversibility index and reported that the time asymmetry of EEG signals is significantly reduced when the brain is lesioned [30]. Furthermore, Yao et al. used the time irreversibility index and reported a nonlinear decrease in EEG signals during seizure-free intervals in individuals with epilepsy [31]. Gadhoumi et al. successfully differentiated between seizure and nonseizure intervals in patients with epilepsy using the time irreversibility index [32]. Yao et al. successfully classified sleep EEG signals using the time irreversibility index [33]. Bernardi et al. reported that compared with that in healthy controls (HCs), the irreversibility of MEG signals in individuals with obsessive–compulsive disorder (OCD) was concentrated at a fast timescale and evenly distributed within the same hemisphere [34]. Fan et al. achieved good classification results when ECG signals were classified using time-irreversible indices based on the permutation Jensen–Shannon distance [35].

By analyzing the application of the time irreversibility index to study electrophysiological time series, we reached the following two conclusions. First, analyzing the time irreversibility index of brain signals can improve our understanding of how the brain processes information via specific neural pathways and temporal sequences, and this directionality aids in understanding how efficiently the brain filters, integrates and understands external stimuli. Second, analyzing the time irreversibility index of brain signals can elucidate the temporal dynamics of the brain during information integration processes owing to the temporal sequencing and irreversibility of interactions among different brain regions. Studying this irreversibility can reveal how the brain coordinates information from different senses in the time dimension to form a complete perceptual experience. We hypothesized that the statistical properties of nonequilibrium features of MEG signals in schizophrenia (SZ) patients at different time scales differed from those of HC. Therefore, we suggest that the time irreversibility indices of the MEG signals of SZ patients can be distinguished from those of HCs. To test our hypothesis, we designed a multiscale permutation time irreversibility index algorithm to study the nonlinear nonequilibrium characteristics of MEGs at different time scales.

## 2. Methods

### 2.1. Participants

In this investigation, 34 participants, including 19 people with schizophrenia (age: 25±8.32, 2 females) and 15 healthy volunteers (age: 25.4±4.85, 3 females), were included. All patients included in this investigation were sourced from the outpatient clinic of the Nanjing Brain Hospital. All healthy volunteers (the HCs in this study) were recruited by verbal invitation from the researchers. After being made aware of the details of the study, all the participants signed an informed consent form. All the participants were required to meet the following conditions: (a) no other psychiatric illnesses, (b) no history of severe traumatic brain injury, (c) demonstration of normal intellectual development, (d) not pregnant, and (e) no record of alcohol or drug abuse during the two weeks before the MEG data were obtained. This study was approved by the Ethics Committee of the Nanjing Brain Hospital (2017-KY015).

### 2.2. Collection of MEGs

MEG recordings were collected by the staff of the Magnetoencephalography Unit of the Nanjing Brain Hospital using a Canadian CTF/VSM 275 full-head superconducting quantum interference device (SQUID)-MEG system (Canadian VSM Company, Coquitlam, BC, Canada). Because metallic zippers, jewelry, and gems can interfere with electromagnetic signals, individuals were required to remove these objects before the MEG recording was performed. When the MEG recordings were being collected, the individuals involved were told to enter an electromagnetically protected room, sit still on a test bed with their eyes open, place their head within a helmet-like sensor array, and remain relaxed. Three small magnetic coils were placed in front of the tip of the subject’s nose, and the openings of both ear canals were used to determine the position of the subject’s head relative to the SQUID sensors. During the MEG data collection process, the data collectors monitored and guided the participants through the cameras and intercoms installed in the electromagnetically shielded room. After confirming that the individuals were in a relaxed state, the MEG recordings were obtained, and the resting-state data were continuously recorded for two minutes. The process of digitizing the recorded MEG data was subsequently carried out at a sampling frequency of 1200 Hz. To facilitate manual checking of the MEG data at a later stage, the ECG and electrooculogram (EOG) signals from each participant were also collected simultaneously. Participants were instructed to remain still throughout the MEG recording session, avoiding any movements, including blinking and muscle contractions, that might have interfered with the accuracy of the data. If the participants made movements that could have affected the accuracy of the research conclusions, the collected signals were abandoned, and new data were collected.

### 2.3. Preprocessing of MEGs

First, the MEG data were manually inspected using the MATLAB EEGLAB 12.0 toolbox to eliminate data with excessive interference. Independent component analysis (ICA) was used to remove other biological noise and artifacts from the MEG recordings. Different independent components are decomposed via ICA. Interference components (ECG and EOG) are identified by combining multi-dimensional features, which are then removed to finally reconstruct clean signals. The FieldTrip toolbox within MATLAB 2013 was subsequently employed to conduct offline preprocessing of the MEG data. The raw data were filtered with a digital low-pass filter with a frequency of 65 Hz, and the power line component at 50 Hz was notch-filtered with the relevant bandstop filter. Finally, the MEG signals were divided into five frequency bands using appropriate bandpass filters: δ (1–4 Hz), θ (5–8 Hz), α (9–12 Hz), β (13–29 Hz), and γ (30–65 Hz).

### 2.4. Multiscale Permutation Time Irreversibility

The core concept of the multiscale permutation time irreversibility algorithm is to first obtain a coarse-grained version of the original time series to obtain a multiscale time series [36,37] and then to calculate the permutation-based time irreversibility parameters at each scale to determine the multiscale permutation time irreversibility parameters of the original sequence. The detailed calculation process is as follows.

First, a coarse-grained version of the original time series {x(n):n=1,2,…,l} with a scale of *s* is obtained via Formula (1), yielding $\left\{x_{s}\left(N\right):N=1,2,\ldots ,\left\lfloor l/s\right\rfloor \right\}$, where *l* is the length of the original time series.

(1) $$\begin{array}{c} x_{s}\left(N\right)=\frac{1}{s}\sum_{n=s(N-1)+1}^{Ns}x(n)\left(1\leq N\leq \left\lfloor l/s\right\rfloor \right) \end{array}$$

With an embedding dimension of *m* and an embedding delay of τ, phase space reconstruction is subsequently performed on the basis of the coarse-grained time series $x_{s}\left(N\right)$ according to Formula (2) to obtain ${X_{s}}_{m}^{\tau }\left(t\right)$. The optimal values of the embedding dimension *m* and embedding delay τ can be set according to the false nearest neighbors (FNN) [38] and C-C [39] algorithms.

(2) $$\begin{array}{c} {X_{s}}_{m}^{\tau }\left(t\right)=\left\{x_{s}\left(t\right),x_{s}\left(t+\tau \right),\ldots ,x_{s}\left(t+\left(m-1\right)\tau \right)\right\} \end{array}$$

Afterward, the embedding vector ${X_{s}}_{m}^{\tau }\left(i\right)=\left\{x_{s}\left(i_{1}\right),x_{s}\left(i_{2}\right),\ldots ,x_{s}\left(i_{m}\right)\right\}$ in phase space is arranged in ascending order, and the arranged sequence is $X_{s}\left(j\right)=\{x_{s}\left(j_{1}\right),x_{s}\left(j_{2}),\ldots ,x_{s}\left(j_{m}\right)\right\}$. If the vector contains elements with equal values, they are arranged according to the order in which they appear, and their coordinates are modified to the minimum value of the elements in the same group. To directly reflect the spatial structural characteristics of the vector, the positions $j_{i}$ of the original vector elements in the permuted vector $X_{s}\left(j\right)$ are used to form the amplitude permutation $f\left(\pi_{k}\right)=\left(j_{1},j_{2}\ldots ,j_{m}\right)$, subsequently obtaining the backward permutation $b(\pi_{k})$ of $f(\pi_{k})$. The probability $p(f(\pi_{k}))$ of $f(\pi_{k})$ and the probability $p(b(\pi_{k}))$ of its backward permutation $b(\pi_{k})$ are calculated, and the permutation entropy and the permutation time irreversibility with scale *s* are defined as shown in Formulas (3) and (4), respectively, where *U* denotes the maximum number of forward permutations and *V* denotes the sum of the number of forward permutations and backward permutations. Calculating the permutation entropy and permutation time irreversibility at each scale factor yields the multiscale permutation entropy (MsPE) and multiscale permutation time irreversibility (MsPTIRR) of the original time series, respectively.

(3) $$\begin{array}{c} PE_{s}=-\sum_{k=1}^{U}p(f(\pi_{k}))\log\left(p\left(f(\pi_{k})\right)\right) \end{array}$$

(4) $$\begin{array}{c} PTIIR_{s}=\sum_{k=1}^{V}p(f(\pi_{k}))\frac{p\left(f(\pi_{k})\right)-p\left(b(\pi_{k})\right)}{p\left(f(\pi_{k})\right)+p\left(b(\pi_{k})\right)} \end{array}$$

### 2.5. Statistical Methods

All the statistical analyses were performed using Statistical Product and Service Solutions (SPSS) software (SPSS25). Kolmogorov-Smirnov tests and F tests were conducted to test whether the data met the assumptions of a normal distribution and homogeneity of variance, respectively. An independent samples t test was adopted to explore significant differences in the MsPTIRR indices between the patients with schizophrenia and HCs. Two-factor analysis of variance was used for results from different frequency bands and different brain regions under a single scale factor.

## 3. Results

The optimal embedding dimension and embedding delay for each subsequence of the MEG data of all individuals were obtained via the FNN algorithm and C-C algorithm. The final *m* and τ were set to 3 and 2, respectively, according to their modes. The permutation time irreversibility and permutation entropy values of the 275 subsequences of the MEG recordings of each participant at each scale condition were computed separately, and their average values were subsequently taken as the permutation time irreversibility index and permutation entropy index for that participant at the given scale condition. The two indices were subsequently averaged for all SZ patients and HCs to obtain the permutation time irreversibility and permutation entropy indices of the patient and HC groups under the given scale conditions. Multiscale analyses were performed for scale factors ranging from 1 to 100, with the scale factor *s* increasing by 1.

**Figure 1:**
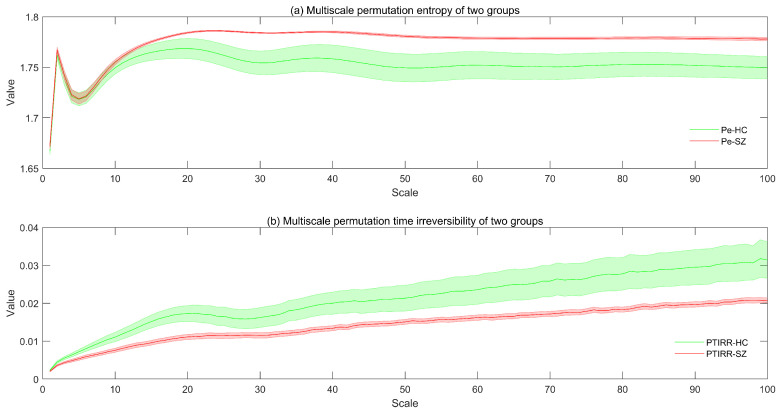
Distributions of the MsPE and MsPTIRR indices for the patients and HCs. The colored lines in the figure represent the mean values of the MsPE and MsPTIRR indices for the patients and HCs, and the shading of the lines represents the standard errors. In (**a**), Pe-HC and Pe-SZ denote the MsPE values for the patients and HCs, respectively. In (**b**), PTIRR-HC and PTIRR-SZ represent the MsPTIRR indices of the patients and HCs, respectively.

The distributions of the MsPE and MsPTIRR indices for the patients and HCs are shown in Figure 1a and Figure 1b, respectively.

As shown in Figure 1a, as the scale factor increases, the MsPE values of the SZ and HC groups first increase, then decrease, then increase again and finally stabilize. When the scaling factor was greater than 10, the MsPE values of the two groups differed, with the values of the SZ group being greater than those of the HC group. This figure also shows that the standard error of the MsPE value of the SZ group was greater than that of the HC group. Moreover, as shown in Figure 1b, the MsPTIRR indices of both groups increase slowly with increases in the scale factor. In addition, the standard error of this indicator was greater in the HC group than in the SZ group. However, the MsPTIRR index of the HC group decreased when the scale factor was in the range of 20–30. Additionally, when the scale factor was increased to 2, the MsPTIRR index of the HC group was greater than that of the SZ group. These results suggest that the MEG data of the SZ group are highly complex with low nonequilibrium. This occurs because the brain activities of SZ patients are disorganized; therefore, the various oscillations are in a somewhat chaotic state and entangled, thus increasing the complexity and decreasing the nonequilibrium of the MEG signals in patients.

**Figure 2:**
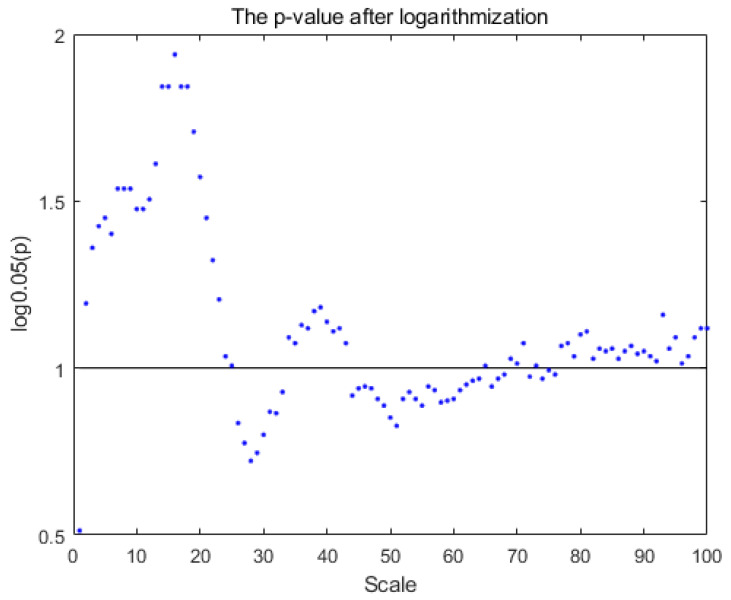
Distribution of the *p* values after logarithmic transformations at different scale factors. The y-axis displays the logarithm of *p* with a base of 0.05, and the horizontal black line indicates a *p* value of 0.05.

Furthermore, the MsPE and MsPTIRR indices differed at various scale factors, suggesting that the dynamics of MEG data have obvious multiscale characteristics; therefore, the dynamics are different at various time scales. A comparison of the trends of the MsPE and MsPTIRR indices at different scale factors in the SZ and HC groups revealed that the MsPTIRR index is more suitable for mining abnormal nonlinear features in the MEG recordings of the SZ group. Therefore, we used a two-tailed independent-samples t test to statistically analyze the MsPTIRR indices of the two groups, and the distributions of the *p* values of the t tests for different scale factors are shown in Figure 2. As shown in Figure 2, the differences in the MsPTIRR indices of the SZ and HC groups differed at different scale factors. The difference in the MsPTIRR index between the two groups was greatest at a scale factor of 16 (p=0.003).

**Figure 3:**
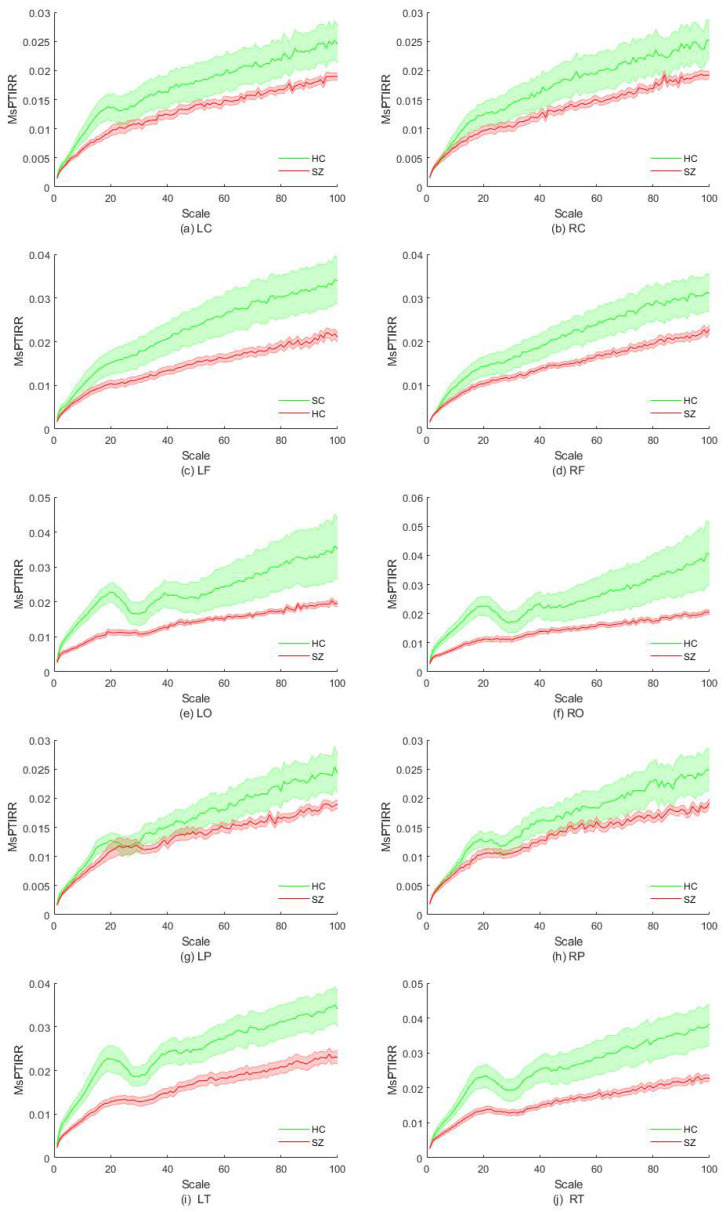
Distribution of the MsPTIRR indices in different brain regions. HC indicates healthy controls, and SZ indicates schizophrenia patients. L represents the left hemisphere of the brain, R represents the right hemisphere of the brain, C denotes the central region, F represents the frontal region, O represents the occipital region, P represents the parietal region, and T represents the temporal region.

In this section, we explore the differences in the MsPTIRR indices of the MEG signals of SZ patients and HCs in different brain regions. First, the brain was divided into two hemispheres, left and right; subsequently, each hemisphere was divided into five brain regions: the central, frontal, parietal, occipital, and temporal regions. The distributions of the MsPTIRR indicators within the different brain regions for SZ patients and HCs are shown in Figure 3. As shown in Figure 3, the overall trends of the MsPTIRR indices in different brain regions in SZ patients and HCs were approximately the same. In addition, as shown in Figure 3, the MsPTIRR indices of the brain regions in the left hemisphere of the SZ patients and HCs were slightly greater than those of the brain regions in the right hemisphere. This is because there is some degree of asymmetry in the structures of the left and right hemispheres of the human brain. Careful observation of the curves in Figure 3 reveals some differences in the trends of the MsPTIRR indices in different brain regions in SZ patients and HCs. In particular, the differences in the MsPTIRR indices between the two groups were greater in the frontal, occipital, and temporal regions than in the central and parietal regions.

The statistical results revealed a statistically significant difference in the MsPTIRR indices of the two groups within the central region of the left hemisphere at scale factors of 15–20, whereas no significant differences in the MsPTIRR indices of the two groups were found within the central region of the right hemisphere at any scale. These results are consistent with the results in Figure 3a,b. In the frontal regions of the left hemisphere, the MsPTIRR indices of the two groups significantly differed as the scale factor increased to 43, whereas in the frontal regions of the right hemisphere, the MsPTIRR indices of the two groups significantly differed only when the scale factor exceeded 75. The significant differences in the MsPTIRR indices of the two groups within these two brain regions increased with increasing scale factors. Furthermore, in the occipital region of the left hemisphere, there was a significant difference in the MsPTIRR indices of the two groups when the scale factor ranged from 2 to 27 and from 34 to 45. The greatest difference in the MsPTIRR indices of the two groups was observed at a scale factor of 16 (p<0.0001). The greatest differences in the MsPTIRR indices of the two groups in the occipital region of the right hemisphere were obtained for the scale factor ranges of 2–26 and 37–41. Furthermore, no statistically significant differences in the MsPTIRR indices of the two groups were found in the parietal regions of the left or right hemisphere at any scale factor. Moreover, in the temporal region of the left hemisphere, significant differences in the MsPTIRR indices of the two groups were found for scale factors greater than 2. However, in the temporal region of the right hemisphere, no significant difference in the MsPTIRR indices of the two groups was found at a scale factor of 28–32. The detailed statistics are shown in Table 1. In addition, the results of the ANOVA showed that the differences between brain regions in the frontal, occipital and temporal regions in the patient group and other brain regions were significantly stronger.

**Figure 4:**
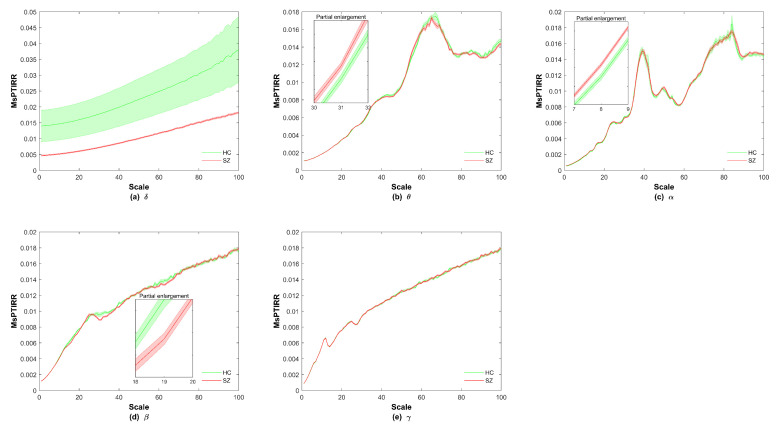
Distribution of the MsPTIRR indices in different frequency bands. HC indicates healthy controls, and SZ indicates schizophrenia patients.

**Figure 5:**
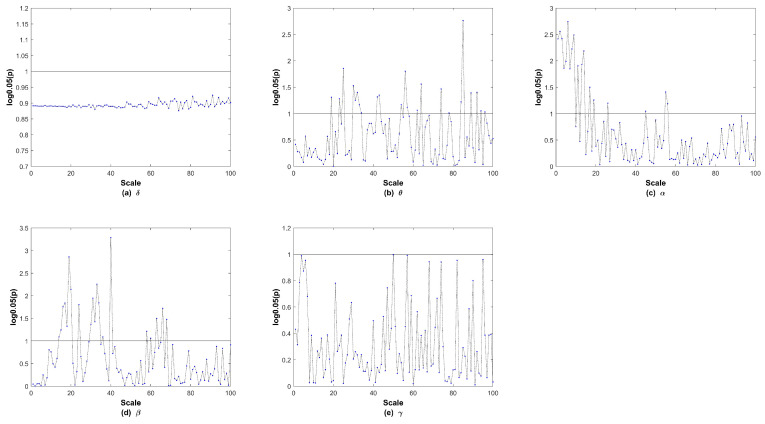
The *p* values in each frequency band after logarithmic transformations. In the subgraph, the y-axis displays the logarithm of *p* with a base of 0.05, and the horizontal black line indicates a *p* value of 0.05.

We next explored the MsPTIRR indices of the MEG data of the two groups in different frequency bands. The distributions of the MsPTIRR indices of the MEG data of both groups in the δ, θ, α, β, and γ bands are shown in Figure 4, and the statistical results for each frequency band are shown in Figure 5. As shown in Figure 4, the MsPTIRR indices of the MEG signals of the two groups increased with increasing scale factor in the δ, β and γ bands. However, in the θ and α bands, as the scale factor increased, the MsPTIRR indices of the two groups showed complex change trends. Instead of only increasing, the values changed considerably, particularly in the α band. Moreover, the MsPTIRR indices of the SZ group were greater than those of the HC group for several scale factors in the θ and α bands. This result occurred owing to the highly abnormal α and θ oscillations that occurred in the resting state in the SZ patients. As shown in Figure 4, the change trends of the MsPTIRR indices in SZ patients and HCs were similar in each frequency band. However, as shown in Figure 5, as the scale factor increased, significant differences in the MsPTIRR values emerged between the two groups in the θ, α, and β bands. The difference is particularly noticeable in the α frequency band when the scale factor is between 2 and 17. In addition, the results of the ANOVA show that the differences between the θ and α bands and other frequency bands in patients are greater than those between other frequency bands.

## 4. Discussion

In this investigation, we explored the nonlinear nonequilibrium characteristics of MEG data in SZ patients using the proposed multiscale permutation time irreversibility algorithm. Overall, the findings revealed that the MsPTIRR indices of the MEG data of SZ patients were lower than those of HCs. The brain region study results revealed significant differences in the MsPTIRR indices of SZ patients and HCs, mainly in the frontal, occipital and temporal regions. The cross-frequency study results revealed significant differences in the MsPTIRR indices between SZ patients and HCs, mainly in the θ, α and β bands. An in-depth discussion of these results is presented below.

First, our results show that the MsPTIRR indices of the MEG data of SZ patients are significantly lower than those of HCs. It demonstrates that alterations have occurred in the dynamic characteristics of oscillatory signals in the cerebral nervous system of SZ patients [40,41]. These findings suggest that, normally, MEG signals in the human brain exhibit different characteristics for forward and reverse processes on the time axis. The transmission of information between neurons in the brain is purposeful and sequential. The individual neuronal components involved in this information transfer process are highly sequential and cannot be easily altered. This is because the human brain must process information via specific neural pathways and time sequences to integrate and categorize relevant information effectively, thus preventing confusion during information processing. Even in the resting state, brain activity is more dynamic in healthy individuals, with frequent neurotransmitter release and neuronal excitation–inhibition regulation; thus, the MsPTIRR index tends to be greater. However, the brains of patients with schizophrenia are characterized by highly disordered states, with reduced brain activity and enhanced synchronization between neurons, resulting in lower MsPTIRR indices. Related studies have shown abnormalities in the synchronization of neurons in the brains of SZ patients [42,43,44].

There are several possible causes of this abnormal neuronal synchronization. (1) SZ patients have imbalances in neurotransmitter levels in the brain, mainly involving dysregulation of the glutamatergic system and abnormalities in the dopaminergic system. There is much evidence that N-methyl-D-aspartate (NMDA) receptors function abnormally in SZ patients, resulting in dysfunction of the glutamatergic system and imbalanced excitation–inhibition in the cortex, leading to abnormal neuronal synchronization [45,46,47,48,49]. The results of this study indicate that dopaminergic neuronal activity is abnormal in SZ patients, affecting the normal discharge of neurons, thereby interfering with normal signaling between neurons and ultimately leading to abnormal neuronal synchronization across the brain [50,51,52,53]. (2) Abnormalities in brain structure and function can also lead to abnormalities in neuronal synchronization. Cerebral white matter fiber tracts are involved in neural pathways that connect different brain regions, and their integrity is crucial for achieving neuronal synchronization. Previous findings suggest that the myelination of white matter fibers is impaired in the brains of SZ patients [54,55,56]. Myelin acts as an insulating layer that wraps around nerve fibers and accelerates nerve signal transmission. Myelination damage slows nerve signaling, attenuates signals, and affects information exchange between different brain regions, leading to abnormal neuronal synchronization. Altered functional connectivity of brain networks can also lead to abnormal neuronal synchronization. Previous studies suggest that people with SZ have abnormal functional connectivity in the brain, leading to altered connections between brain regions, which can impair signal transmission between these regions, in turn leading to abnormal neuronal synchronization [57,58,59,60]. (3) Neurodevelopmental abnormalities can also disrupt neuronal synchronization. Problems with neuronal migration and differentiation during the early stages of brain development can interfere with the normal formation of neural circuits in the brain. Disordered neuronal migration and differentiation in the fetus or during early childhood can result in neurons failing to migrate to their intended locations in the brain or connections between neurons not being established properly, leading to abnormal neuronal synchronization in these brain regions in adults with schizophrenia [61,62]. Furthermore, abnormal synaptic pruning in SZ patients, especially during critical periods such as puberty, disrupts neuronal connectivity patterns in the frontal, occipital, and temporal lobe regions, ultimately leading to abnormal neuronal synchronization [63,64].

In addition, significant differences in the MsPTIRR indices of the two groups were found only in the frontal, occipital and temporal regions. These findings suggest that the abnormal features discussed earlier are more prominent in these three brain regions. The findings of the brain region analysis do not suggest that the abnormal features discussed above are not present in the central or parietal regions of the brains of patients with schizophrenia; rather, they are present less prominently. Our previous study revealed that the difference in MEG complexity between the two groups was particularly prominent in the frontal, occipital and temporal lobe regions, which is consistent with the findings of the present study [65]. Additionally, previous researchers have reported a variety of abnormal activities in the frontal lobe [66,67], occipital lobe [68,69], and temporal lobe [70,71], supporting our findings.

The frequency band analysis revealed differences in the nonequilibrium features of the MEG signals between the patients and HCs, mainly in the θ, α and β bands. This is because α waves are dominant in the resting state, and the α band in the MEG recordings is closer to the θ and β bands than to the δ and γ bands. Our findings are consistent with the findings of several previous studies. Koshiyama et al. reported abnormal phase discontinuities in θ and α waves in schizophrenia patients [72]. Arora et al. identified potential markers of schizophrenia in EEG signals in the θ, α, and β bands [73]. Furthermore, Ramsay et al. revealed that the strength and consistency of neural oscillations in schizophrenia patients were significantly reduced in the θ, α, and β bands [74]. Lin et al. reported that θ and α band power was significantly increased in schizophrenia patients, whereas θ band power was significantly increased and β band power was decreased in HCs [75]. These results support the reliability of our findings.

We successfully identified abnormal nonlinear nonequilibrium (directionality on the time axis) features in the MEG data of SZ patients, and these features could serve as potential markers of schizophrenia. Our findings provide a quantifiable and objective foundation for the clinical diagnosis of schizophrenia, which could increase the accuracy of the diagnosis of this disorder. However, this study has several limitations that should be considered. First, the number of schizophrenia patients included in the study was small. The findings should be validated, and the impact of confounding factors such as age and gender on the results should be considered with a larger sample size. In addition, the nonequilibrium characteristics of MEG signals in SZ patients have only been studied at the sensor level, with no research conducted in the MEG source space. Third, only abnormal nonequilibrium features in the MEG recordings of SZ patients and HCs were investigated, and owing to the small sample size, these features were not utilized in combination with relevant deep learning methods for classification analyses. Therefore, it is necessary to conduct relevant research in the MEG source space in future studies. The findings could be further validated in future studies by collecting more data with a larger sample to enhance their reliability. When substantial data are collected, the identified features can be combined with deep learning methods to conduct recognition studies of schizophrenia. Finally, the most suitable parameters of the MEG recordings could be used as markers of schizophrenia.

## 5. Conclusions

In conclusion, we combined the time irreversibility algorithm with a multiscale time series algorithm and proposed the MsPTIRR algorithm. We used the proposed algorithm to study the nonequilibrium features of the MEG recordings of SZ patients. Comparative analyses revealed that the MsPTIRR index is more suitable than the MsPE for mining nonlinear features in the MEG data of SZ patients. The results revealed that the MsPTIRR indices of the MEG signals of SZ patients were significantly lower than those of HCs for various time scale factors. Thus, schizophrenia appears to disturb the nonequilibrium characteristics of the nervous system. The brain region analysis results revealed significant differences in the nonequilibrium characteristics of MEG signals between SZ patients and HCs at various time scales in the frontal, occipital and temporal lobe regions. Moreover, the analysis of different frequency bands revealed significant differences in the nonequilibrium characteristics between the two groups, which were present mainly in the θ, α and β bands. Abnormal nonequilibrium features in MEG signals found at various time scales using the MsPTIRR method may be potential markers of schizophrenia in MEG recordings and may provide an objective basis for the clinical diagnosis of this disorder.

## Figures and Tables

**Table 1:** Summary of t test results in different brain regions.

Brain Regions	Scale Intervals with Significant Differences
LC	[15,20]
RC	No significant difference
LF	[43,100]
RF	[75,100]
LO	[2,27] and [34,45]
RO	[2,26] and [37,41]
LP	No significant difference
RP	No significant difference
LT	[2,100]
RT	[2,27] and [32,100]

## Data Availability

The data in this paper may be requested from the corresponding author upon reasonable request.
